# Supplementary material for: Relationship between Dietary Inflammatory Index and Postpartum Depression in Exclusively Breastfeeding Women
Source: Nutrients. 2022 Nov 25;14(23):5006. doi: 10.3390/nu14235006 (PMC9738724; doi:10.3390/nu14235006)
Supplement: Supplementary file 1 [file nutrients-14-05006-s001.zip › nutrients-2027178-supplementary.pdf]

Relationship between Dietary Inflammatory Index and Postpartum Depression in Exclusively Breastfeeding Women

Supplementary Materials:

Table S1: Dietary intakes of breastfeeding mothers across tertiles of the DII

Nutrients	DII Tertiles (Mean ± SD)			*P* value[1]
	Q1	Q2	Q3	
Energy(kcal/d)	2271.93±545.34	1971.26±339.55	1784.06±479.47	＜0.001*
Protein(g/d)	92.91±28.27	74.38±16.25	59.62±20.40	＜0.001*
Fat(g/d)	85.21±20.07	87.33±12.79	99.55±24.67	＜0.001*
Carbohydrate(g/d)	283.45±95.68	252.52±71.49	230.35±90.38	＜0.001*
Cholesterol(mg/d)	499.32±234.99	663.01±338.93	711.58±299.85	＜0.001*
Dietary Fiber(g/d)	12.99±4.83	8.14±2.38	4.74±2.29	＜0.001*
Vitamin A(μg RE/d)	658.76±265.50	406.45±146.82	275.57±112.19	＜0.001*
Vitamin B1(mg/d)	0.81±0.24	0.62±0.13	0.48±0.15	＜0.001*
Vitamin B2(mg/d)	1.49±0.58	1.16±0.38	0.90±0.46	＜0.001*
Niacin(mg/d)	23.39±7.41	18.44±4.27	15.68±4.68	＜0.001*
Vitamin C(mg/d)	126.69±58.50	65.79±25.21	37.03±20.37	＜0.001*
Vitamin E(mg/d)	18.80±8.38	14.02±3.18	9.42±2.65	＜0.001*
Iron(mg/d)	22.82±7.32	17.46±5.15	12.76±5.08	＜0.001*
Zinc(mg/d)	10.98±3.38	8.17±1.81	6.18±2.02	＜0.001*
Saturated fatty acid(g/d)	36.34±9.72	36.34±5.98	41.05±10.34	＜0.001*
Monounsaturated fatty acids(g/d)	43.49±10.32	38.09±5.81	37.90±8.56	＜0.001*
Polyunsaturated fatty acids(g/d)	11.93±7.06	9.22±1.94	8.38±1.97	＜0.001*
Folate(μg/d)	446.64±151.07	318.54±80.10	204.54±144.16	＜0.001*

DII, Dietary inflammation index.

1 One-way ANOVA for continuous variables.

* *P*＜0.05.

Table S2: Risk factors associated with PPD among exclusively breastfeeding women (n= 293)

	Variables	β	Wald x^2	OR(95%CI)	*P* value[1]
Crude Model	DII groups				0.024*
	Q1 VS Q3	-0.66	4.69	0.52(0.29, 0.94)	0.030*
	Q2 VS Q3	-0.78	6.66	0.46(0.25, 0.83)	0.010*
Model1	Age groups (years)				0.344
	≤ 25 VS 36 ~ 45	-0.19	0.09	0.83(0.25, 2.74)	0.759
	26 ~ 35 VS 36 ~ 45	0.32	0.41	1.381(0.51, 3.70)	0.522

	DII groups				0.016*
	Q1 VS Q3	-0.69	4.59	0.50(0.27, 0.94)	0.032*
	Q2 VS Q3	-0.90	7.77	0.406(0.21, 0.77)	0.005*
	Educational levels				0.004*
	Junior high school and below VS Postgraduate and above	2.54	4.72	12.69(1.28, 125.69)	0.030*
	Specialized degree VS Postgraduate and above	1.00	5.35	2.72(1.17, 6.36)	0.021*
	Bachelor's degree VS Postgraduate and above	0.17	0.17	1.186(0.520, 2.70)	0.685
	Occupational levels Unemployed VS Employed	0.71	6.39	0.49(0.29, 0.85)	0.011*
	Number of children 1 VS ≥ 2	0.65	5.62	1.91(1.12, 3.26)	0.018*
Model 2	Age groups(years)				0.413
	≤25 VS 36~45	0.03	0.00	1.03 (0.29, 3.67)	0.963
	26~35 VS 36~45	0.46	0.73	1.584(0.55, 4.55)	0.392
	DII groups				0.013*
	Q1 VS Q3	-0.75	4.72	0.47 (0.24, 0.93)	0.030*
	Q2 VS Q3	-0.98	8.12	0.38(0.19, 0.74)	0.004*
	Educational levels				0.031*
	Junior high school and below VS Postgraduate and above	2.38	3.84	10.84(1.00, 117.56)	0.05*
	Specialized degree VS Postgraduate and above	0.85	3.28	2.341(0.93, 5.88)	0.07
	Bachelor's degree VS Postgraduate and above	0.17	0.15	1.187(0.49, 2.86)	0.703
	Occupational levels Unemployed VS Employed	-0.74	6.19	0.48(0.27, 0.86)	0.013*
	Number of children 1 VS ≥ 2	0.74	6.27	2.094(1.17, 3.74))	0.012*
	Number of caregivers				0.025*
	1 VS ≥ 3	1.37	7.38	3.93(1.46, 10.55)	0.007*
	2 VS ≥ 3	0.68	3.15	1.98(0.93, 4.19))	0.076
	PSSS score levels Low to medium social support VS	0.64	4.43	1.893(1.05, 3.43)	0.035*

High social support<br>PSQI score levels<br>Good sleep quality VS Moderate or poor sleep quality	-0.75	6.64	0.47 (0.27, 0.84)	0.010*

PSSS, Perceived Social Support Scale. PSQI, Pittsburgh Sleep Quality Index. DII, dietary inflammatory index. PPD, postpartum depression.

1 Binary logistic regression.

Crude Model：Unadjusted for any covariate.

Mode 1：Adjusted age(≤ 25, 26 ~ 35, 36 ~ 45), educational levels (junior high school and below, specialized degree, bachelor's degree, postgraduate and above), occupational levels (employed, unemployed), number of children (1, ≥ 2).

Model 2: Adjusted for age(≤ 25, 26 ~ 35, 36 ~ 45), educational levels (junior high school and below, specialized degree， bachelor's degree, postgraduate and above), occupational levels (employed, unemployed), number of children (1, ≥ 2), number of postpartum caregivers (1, 2, ≥ 3), PSSS score level (low to medium social support, high social support) and PSQI score level (good sleep quality, moderate or poor sleep quality).

* $P < 0.05$.
